# Seasonal Variation of Microbial Diversity of Coastal Sediment in Tongyeong, South Korea, Using 16S rRNA Gene Amplicon Sequencing

**DOI:** 10.1128/MRA.00446-21

**Published:** 2021-07-08

**Authors:** Nur Indradewi Oktavitri, Jong-Oh Kim, Kyunghoi Kim

**Affiliations:** a Department of Ocean Engineering, Pukyong National University, Busan, Republic of Korea; b Environmental Engineering, Faculty of Science and Technology, Universitas Airlangga, Surabaya, Indonesia; c Institute of Marine Biotechnology, Pukyong National University, Busan, Republic of Korea; Montana State University

## Abstract

Benthic microbial diversity in Tongyeong, South Korea, was analyzed using next-generation sequencing of the 16S rRNA genes, to reveal the effects of seasonal variations on the microbial community in sediment. *Proteobacteria* was the dominant phylum, with a relative abundance of 61.5 to 68.1%.

## ANNOUNCEMENT

Predicting the distribution and abundance of bacteria as a response to ecosystem changes is important (1). Suh et al. (2) reported that the changing of the seasons affects the abundance of microbes in the water mass in the South Sea of South Korea. Microbial classes in the water masses in winter and spring were different than those in summer and autumn (2).

The conditions in the semienclosed bay at Tongyeong, South Korea, are interesting to study; the water is shallow, and the water exchange is slow (3). Seasonal effects on the semienclosed bay cause problems such as red tide and hypoxia (4). Although environmental problems in Tongyeong Bay are serious, studies on the microbial diversity in sediment are lacking. An exploration of the taxonomic diversity with high-throughput pyrosequencing techniques showed the distribution and abundance of marine bacteria (2). Thus, we investigated the microbial diversity of Tongyeong Bay sediment through the sequencing of 16S rRNA genes by using high-throughput sequencing.

Sampling was conducted in April, August, October, and December 2019, to represent seasonal data for spring, summer, autumn, and winter, respectively. Sediments were collected at a 20-cm depth from the surface of the sediment in Tongyeong Bay (34^°^47.4420′N, 128^°^25.5700′E) using a grab sampler. The sediment samples were immediately transferred to the laboratory using an ice box at 4°C and were stored at −20°C until DNA extraction. Samples were homogenized, and total genomic DNA was extracted using the DNeasy PowerMax soil kit (Qiagen). Library preparation was performed with the Herculase II Fusion DNA polymerase and Nextera XT index kit v2, using Bakt_341F and Bakt_805R primers (5, 6) to amplify the V3 to V4 region of the 16S rRNA gene. The prepared libraries were sequenced using the Illumina MiSeq platform at Macrogen, Inc. (South Korea). Raw reads were paired-end merged using FLASH v1.2.11 (7). Denoising strategies were applied to obtain amplicon sequence variants (ASVs) by Divisive Amplicon Denoising Algorithm 2 (DADA2) v1.16.1 (8) in R v4.0. Before the denoising analysis, both primer sequences were removed using Cutadapt (9). The standard processing steps in the DADA2 workflow were performed, including quality filtering [maxEE = c(2,5)], dereplication, learning the data set-specific error model, ASV inference, and chimera removal. The naive Bayesian classifier (10) method was implemented for taxonomic assignment using the Ribosomal Database Project (RDP) training set 18 database (11).

**FIG 1:**
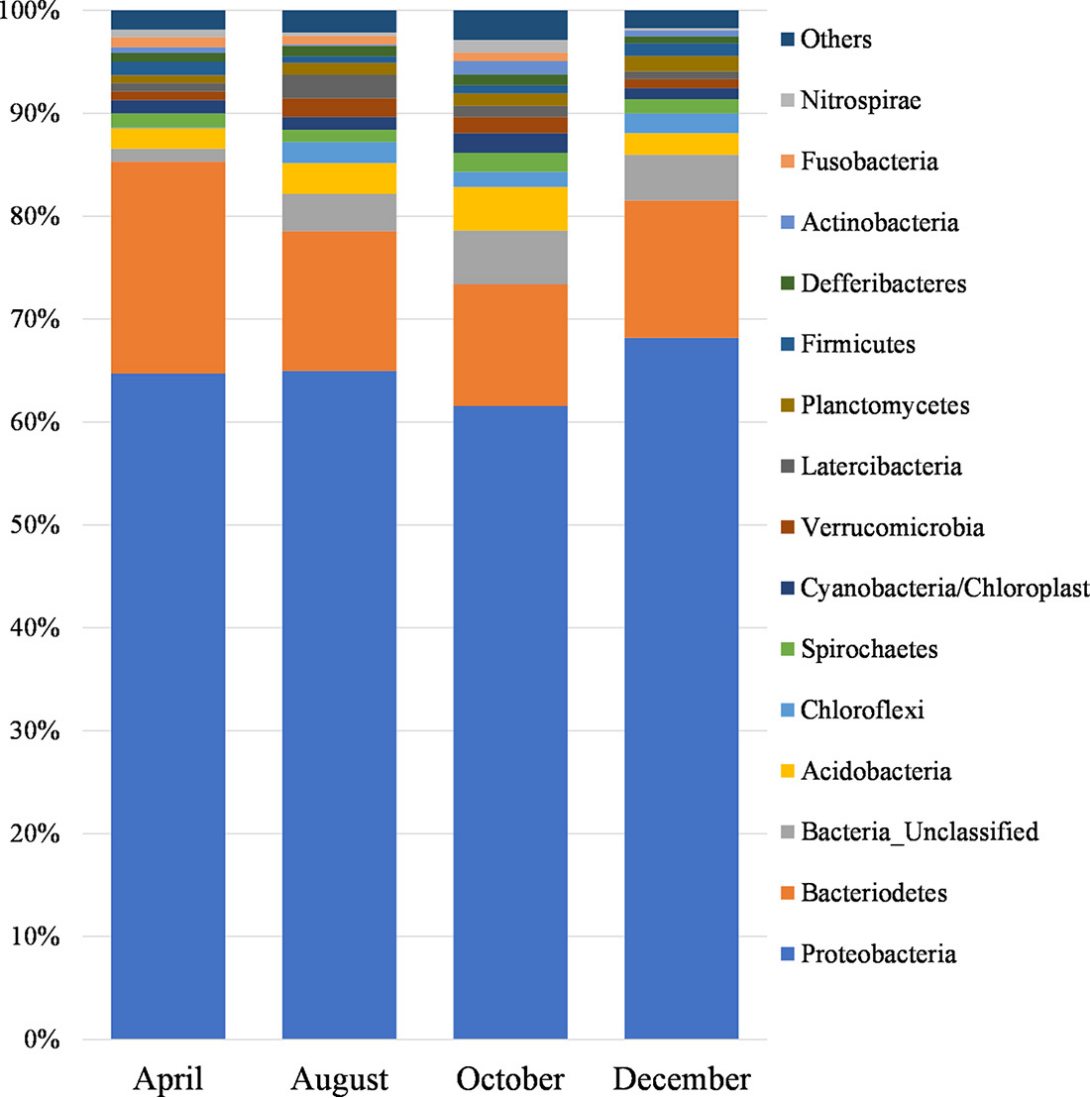
Top 15 microbial phyla in relative abundance in different months. Each color represents a different phylum.

A description of the bioinformatic process is presented in Table 1. Analysis of the microbial diversity in Tongyeong Bay showed that *Proteobacteria* was the most abundant phylum, with a relative abundance of 61.5 to 68.1%, followed by *Bacteroidetes* (11.8 to 20.6%), unclassified bacteria (1.3 to 5.2%), and *Acidobacteria* (2.0 to 4.3%). (Fig. 1). *Proteobacteria* contributed more than one-half of the biomass of bacterial phyla in most surface marine sediments (12). *Proteobacteria* played a role in nutrient and organic matter decomposition in eutrophicated (13, 14) and polluted marine fish farm (15) sediments. Our results provide useful information on the microbial community and may facilitate environmental remediation for Tongyeong Bay.

**TABLE 1:** Summary description of the bioinformatic process

Month	No. of input reads	No. of filtered reads	No. of denoised forward reads	No. of denoised reverse reads	No. of merged reads	No. of nonchimeric reads
April	174,306	95,893	71,036	68,274	22,055	11,257
August	182,753	112,555	86,452	88,083	33,611	16,633
October	116,328	97,399	70,867	78,484	29,823	18,482
December	112,631	90,984	69,757	74,367	29,917	18,106

### Data availability.

The 16S rRNA gene amplicon sequences obtained in this study have been deposited in the NCBI Sequence Read Archive (SRA) under the accession number PRJNA712535.
